# Clinical Heterogeneity in Autosomal Recessive Bestrophinopathy with Biallelic Mutations in the *BEST1* Gene

**DOI:** 10.3390/ijms21249353

**Published:** 2020-12-08

**Authors:** Karsten Hufendiek, Katerina Hufendiek, Herbert Jägle, Heidi Stöhr, Marius Book, Georg Spital, Günay Rustambayova, Carsten Framme, Bernhard H. F. Weber, Agnes B. Renner, Ulrich Kellner

**Affiliations:** 1 Medizinische Hochschule Hannover, Universitätsklinik für Augenheilkunde, Carl-Neuberg-Straße 1, 30625 Hannover, Germany; hufendiek.katerina@mh-hannover.de (K.H.); framme.carsten@mh-hannover.de (C.F.); 2 Klinik und Poliklinik für Augenheilkunde, Universitätsklinikum Regensburg, Franz-Josef-Strauss-Allee 11, 93053 Regensburg, Germany; herbert.jaegle@ukr.de (H.J.); a.renner@berlin.de (A.B.R.); 3 Institut für Humangenetik, Universität Regensburg, Franz-Josef-Strauss-Allee 11, 93053 Regensburg, Germany; heidi.stoehr@klinik.uni-regensburg.de (H.S.); bweb@klinik.uni-regensburg.de (B.H.F.W.); 4 Augenzentrum am St. Franziskus-Hospital, Hohenzollernring 74, 48145 Münster, Germany; marius.gt.book@gmail.com (M.B.); georg.spital@augen-franziskus.de (G.S.); 5 Zarifa Aliyeva National Ophthalmology Centre, 32/15 Javadkhan Str., Baku AZ 1114, Azerbaijan; gunay.rustambayova@yahoo.com; 6 Institut für Klinische Humangenetik, Universitätsklinikum Regensburg, Franz-Josef-Strauss-Allee 11, 93053 Regensburg, Germany; 7 Augenarztpraxis Regensburg, Hoppestraße 5, 93049 Regensburg, Germany; 8 Zentrum für Seltene Netzhauterkrankungen, AugenZentrum Siegburg, MVZ Augenärztliches Diagnostik- und Therapiecentrum Siegburg GmbH, Europaplatz 3, 53721 Siegburg, Germany; 9 RetinaScience, Postfach 301212, 53192 Bonn, Germany

**Keywords:** autosomal recessive bestrophinopathy (ARB), inherited retinal dystrophy, *BEST1*, bestrophin-1, fundus autofluorescence, optical coherence tomography, phenotyping

## Abstract

Autosomal recessive bestrophinopathy (ARB) has been reported as clinically heterogeneous. Eighteen patients (mean age: 22.5 years; 15 unrelated families) underwent ophthalmological examination, fundus photography, fundus autofluorescence, and optical coherence tomography (OCT). Molecular genetic testing of the *BEST1* gene was conducted by the chain-terminating dideoxynucleotide Sanger methodology. Onset of symptoms (3 to 50 years of age) and best-corrected visual acuity (0.02–1.0) were highly variable. Ophthalmoscopic and retinal imaging defined five phenotypes. Phenotype I presented with single or confluent yellow lesions at the posterior pole and midperiphery, serous retinal detachment, and intraretinal cystoid spaces. In phenotype II fleck-like lesions were smaller and extended to the far periphery. Phenotype III showed a widespread continuous lesion with sharp peripheral demarcation. Single (phenotype IV) or multifocal (phenotype V) vitelliform macular dystrophy-like lesions were observed as well. Phenotypes varied within families and in two eyes of one patient. In addition, OCT detected hyperreflective foci (13/36 eyes) and choroidal excavation (11/36). Biallelic mutations were identified in each patient, six of which have not been reported so far [c.454C>T/p.(Pro152Ser), c.620T>A/p.(Leu207His), c.287_298del/p.(Gln96_Asn99del), c.199_200del/p.(Leu67Valfs*164), c.524del/p.(Ser175Thrfs*19), c.590_615del/p.(Leu197Profs*26)]. *BEST1*-associated ARB presents with a variable age of onset and clinical findings, that can be categorized in 5 clinical phenotypes. Hyperreflective foci and choroidal excavation frequently develop as secondary manifestations.

## 1. Introduction

Mutations in the Bestrophin 1 (*BEST1*, NM_004183.4) gene cause a variety of retinal dystrophies with distinct clinical features including autosomal dominant Best vitelliform macular dystrophy (BVMD, MIM 153700) [1], autosomal dominant vitreo-retinochoroidopathy (ADVIRC, MIM 193220) [2], autosomal dominant MRCS syndrome (microcornea, retinal dystrophy, cataract, posterior staphyloma, MIM 193220) [3], autosomal dominant retinitis pigmentosa (MIM 613194) [4] and autosomal recessive bestrophinopathy (ARB, MIM 611809). The latter is caused by biallelic homozygous or compound heterozygous mutations in the *BEST1* gene [5] and was termed ARB by Burgess et al. [6]. ARB is characterized by visual acuity loss due to multiple yellowish subretinal deposits of various sizes at the posterior pole and in the mid-periphery, intraretinal cystoid spaces, and serous retinal detachment [6]. High hyperopia and a shallow anterior chamber with the risk of angle-closure glaucoma can be associated with ARB [6].

The *BEST1* gene encodes bestrophin-1, a 585 amino acid transmembrane protein located in the basolateral membrane of the retinal pigment epithelium (RPE) [1,7], where it functions as a calcium-activated, volume regulated anion channel [8,9]. A number of properties of bestrophin-1 appear to be essential for the RPE [10,11]. Recently, a better understanding of the molecular pathology of *BEST1*-related phenotypes was achieved by demonstrating that *BEST1* gene defects in BVMD and ARB trigger a strong reduction of *BEST1*-mediated anion transport function compared to control, while ADVIRC mutations cause an increased anion permeability suggesting a stabilized open state condition of channel gating. Furthermore, BVMD and ARB differ by the degree of mutant protein turnover and by the site of subcellular protein quality control with adverse effects on lysosomal pH only in BVMD [12,13] To this end, ARB-associated missense mutations result in the formation of greatly unstable mutant protein subunits that are recognized by the endoplasmic reticulum control machinery and thereby are prone for rapid degradation via the proteasome [12].

Multiple single case reports, few families, and some larger Asian and European cohorts with ARB were reported [14,15,16,17,18]. Here we present a large European study comprising the clinical and functional features of 18 individuals with ARB from 15 unrelated families. In this series, 16 different *BEST1* mutations were identified, while six of them have not been reported previously. Based on detailed retinal imaging five distinct phenotypes were defined, one of them has not been reported previously in association with ARB. In addition, hyperreflective foci and focal choroidal excavation are two features that were seen more frequently than previously reported.

## 2. Results

### 2.1. Patient History

Eighteen patients (nine females, nine males) from 15 unrelated families were examined in this study (Table 1). A first round of examination was done with a mean age of 22.5 years and a wide range between 3 and 50 years. In 15/18 patients (mean 6.8 years; range 1–26 years), one or more follow-up examinations were performed at least one year after the initial examination. The patient-reported mean onset of symptoms was at 12.7 years of age (range 3–50 years).

In 9/15 families only a single family member was reported to be affected. In 2/15 families (F13, F15) family history indicated further affected family members with disease onset in the second decade of life, but they were not available for clinical examination. In 4/15 families additional affected family members could be examined. Disease presentation and age of onset was variable within these 4 families. Accordingly, in one family (F5), two sisters (#5, #6) were affected at the same age, but they presented with different phenotypes. In a second family (F4), two sisters (#4, #12) presented with fundus changes of similar phenotype at the same age of 6 years, but one sister (#12) never developed visual problems until her last examination at 26 years of age. In the third family (F14), two brothers presented with late onset at 46 and 50 years of age (#16, #17), the fundus changes showed different phenotypes in the two eyes of one brother (#16), his left eye showed a similar phenotype as his brother #17 in both eyes. In the fourth family (F2), the paternal uncle of patient #2 had a late onset of BVMD at 40 years of age, while the father was unaffected at 36 years of age (both heterozygous for the c.454C>T mutation).

### 2.2. Clinical Findings

A summary of the clinical findings is presented in Table 1. The majority of patients noted visual problems in childhood, some of them experienced progressive loss of visual function during the course of the disease (#10, #13, #18). Symptoms at onset were frequently blurred vision (13/18) and sometimes night blindness (#10, #15) or photophobia (#1). Three patients (#5, #6, #12) reported no symptoms and presented with bilateral normal visual acuity despite subretinal fluid in the macula including the fovea.

Refractive errors were present in all but one patient (mean +1.89 D; range −3.25 D to +6.00 D) with hyperopia in the majority of cases (14/18). Hyperopic refraction was not directly associated with marked intraretinal cystoid spaces or subretinal fluid on OCT. Astigmatism was mostly mild (mean −1.07 D; range −0.25 D to −3.25 D). Snellen visual acuity was markedly reduced (mean 0.51; range 0.02 −1.0) in 14/18 patients. During follow-up, visual acuity increased notably in the youngest patient (#1) due to prescription of glasses. Small variations of visual acuity were recognized in other eyes, but most patients had some decline of visual acuity during follow-up (mean 0.44; range 0.05–1.0). Visual acuity was similar on both eyes in most patients, marked differences developed in #2, #3, #14, and #16. In the two latter patients, this was due to amblyopia of the left eye.

Anterior segment disorders were observed in 2 patients. Patient #2 showed a bilateral upper lid ptosis. In patient #15 intraocular pressure was moderately increased (RE 23 mmHg; LE 24 mmHg) under therapy for angle-closure glaucoma. Anterior segment was consistent with mild hyperopia and a shallow chamber angle (RE Shaffer grade II, LE Shaffer grade I to II).

### 2.3. Clinical Phenotypes

**Figure 1:**
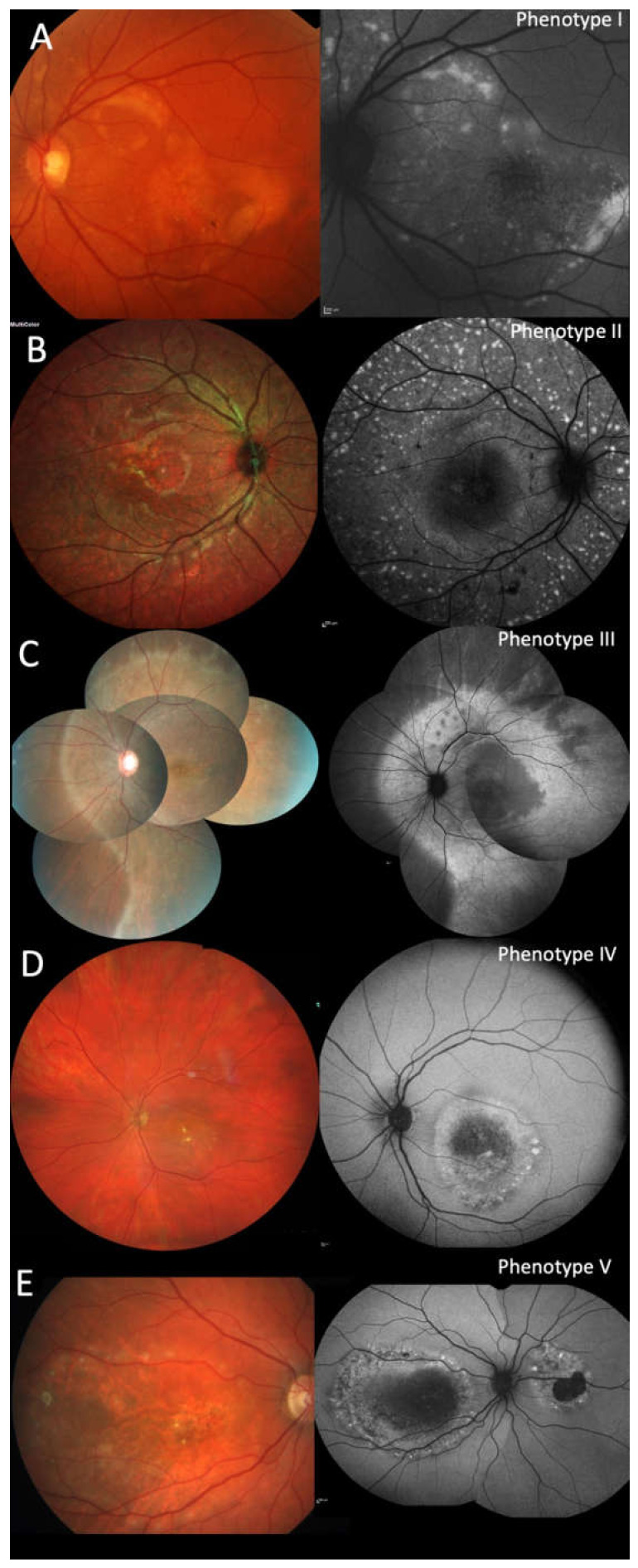
Autosomal recessive bestrophinopathy phenotypes I-V: Fundus photography and fundus autofluorescence (FAF): (**A**). (#5) multiple fleck-like and often confluent yellow lesions involving the posterior pole and extending to the mid-periphery beyond the vascular arcades and nasal of the disc. (**B**) (#8) multiple small fleck-like, mostly non-confluent yellow lesions involving the posterior pole and extending to the far periphery. (**C**) (#15) a continuous yellowish area without flecks extended partly into the far periphery and was bordered by a sharp demarcation line. (**D**) (#11) one Best vitelliform macular dystrophy (BVMD)-like lesion at the posterior pole resembling unifocal BVMD. (**E**) (#18) two well-circumscribed lesions consistent with multifocal BVMD.

The ophthalmoscopic and retinal imaging findings separated the patients into five different phenotypes. The phenotype was similar on both eyes of each patient except for patient #16. The predominant phenotype I (19/36 eyes; #1–5, #7, #10, #12, #16 OS, #17) was characteristic for ARB: multiple fleck-like and often confluent yellow lesions involving the posterior pole and extending to the mid-periphery beyond the vascular arcades and nasal of the disc (Figure 1A). Phenotype II was different (8/36; #8, #9, #13, #14) with multiple small fleck-like, mostly non-confluent, yellow lesions involving the posterior pole and extending to the far periphery (Figure 1B). In one patient (2/36 eyes; #15) a continuous yellowish area without flecks extended partly into the far periphery and was bordered by a sharp demarcation towards the periphery (phenotype III; Figure 1C). More localized lesions resembling BVMD were seen in four patients with either one lesion at the posterior pole (phenotype IV, 2/36; #11; Figure 1D) resembling unifocal BVMD, or two to four well-circumscribed lesions consistent with multifocal BVMD (phenotype V, 5/36; #6, #16 OD, #18; Figure 1E). The phenotypes were similar in at least one eye of the two siblings of two families (#4/#12 and #16/#17), However, in family F5, patient #5 showed the characteristic ARB phenotype I, her sister (#6) presented with phenotype V. Similar phenotypes were seen at different years of age, therefore the different phenotypes cannot be explained as different stages of the same disease. Independent of the phenotype some eyes (14/36) showed a variable number of pigmented spots (#1, #2 OD, #7, #8, #9, #11, #15 OS, #17).

### 2.4. Fundus Autofluorescence

**Figure 2:**
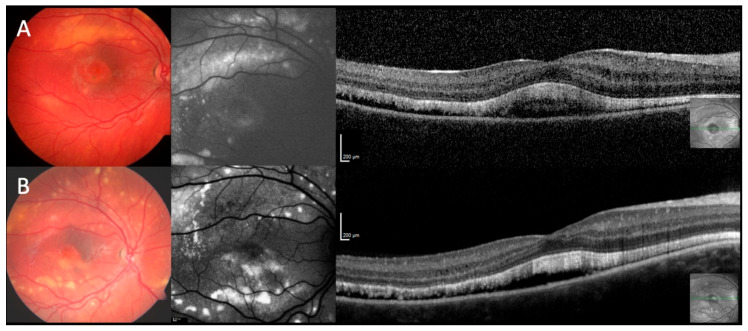
Disease progression. Our youngest patient (#1) (**A**) at the age of 3 (**B**) and 8 years. Fundus photograph at initial visit (**A**, left column) shows multiple yellow subretinal deposits at the posterior pole with accentuation around the vessel arcades. These lesions increased in number and area over time (**B**, left column). Initially, FAF (**A**, middle column) depicts corresponding areas of increased autofluorescence. In some of these areas, FAF intensity increased over time, whereas in other areas FAF intensity decreased with RPE loss (**B**, middle). Optical coherence tomography (OCT; **A**, right column) reveals a marked amount of subretinal fluid and highly reflective material which persist over time (**B**, right column).

As expected for inherited retinal dystrophies, more lesions could be identified by FAF compared to ophthalmoscopy or fundus photography (Figure 1 and Figure 2). Lesions with increased FAF intensity were seen in all phenotypes. Except for phenotype III, the lesions were of variable size and could be isolated flecks, combined flecks such as a chain or comet tail or confluent. Similarly, except for phenotype III, areas of reduced FAF intensity were seen in the majority of eyes (28/36) and always bilaterally. In most eyes there were small spots of reduced intensity, six eyes had developed unilateral larger areas of absent FAF indicating RPE loss (#8–10, #16, #18). In larger progressed lesions increased FAF was usually present at the lower border of the lesion with reduced FAF within the lesion. During follow-up, new lesions evolved in areas with previously normal FAF intensity whereas in some existent lesions FAF intensity decreased corresponding to the degradation of the subretinal deposits (#1, Figure 2 center column). Separate lesions could become confluent and confluent lesions might separate into several lesions. In phenotype III, a large area of homogeneously increased FAF intensity surrounded the posterior pole, partly increased FAF extended along the retinal vessels. There was no change during the two-year follow-up.

### 2.5. Optical Coherence Tomography

**Figure 3:**
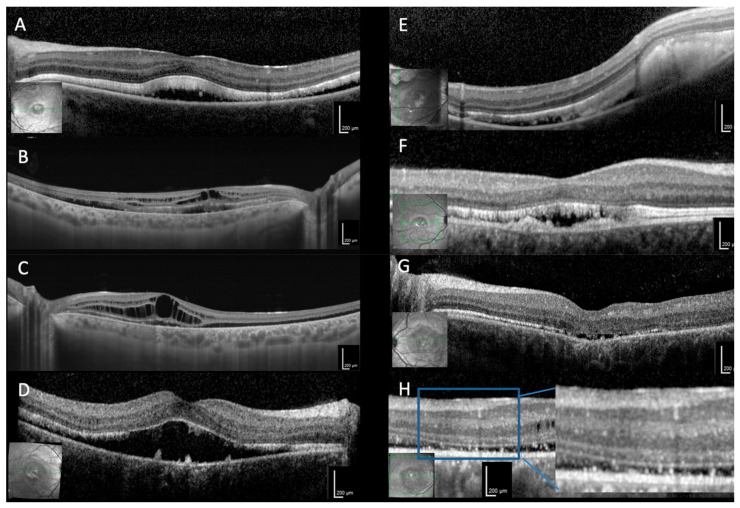
OCT findings. (**A**) Highly reflective material (most likely elongated photoreceptor outer segments) adjacent to the detached retina (#1). (**B**) Moderate (#9, RE) and (**C**) severe (#9, LE) intraretinal cystoid spaces. (**D**) Serous retinal detachment involving the fovea with additional subretinal material adjacent to the RPE presented as bumps (#2). (**E**) The subretinal material was mostly continuous (#7), (**F**) but could also appear as separate stalactites and stalagmites (#12) (**G**) Reduced thickness of the inner retinal layers, temporally the ellipsoid zone is also disintegrated (#2) (**H**) Hyperreflective foci (#15).

**Figure 4:**
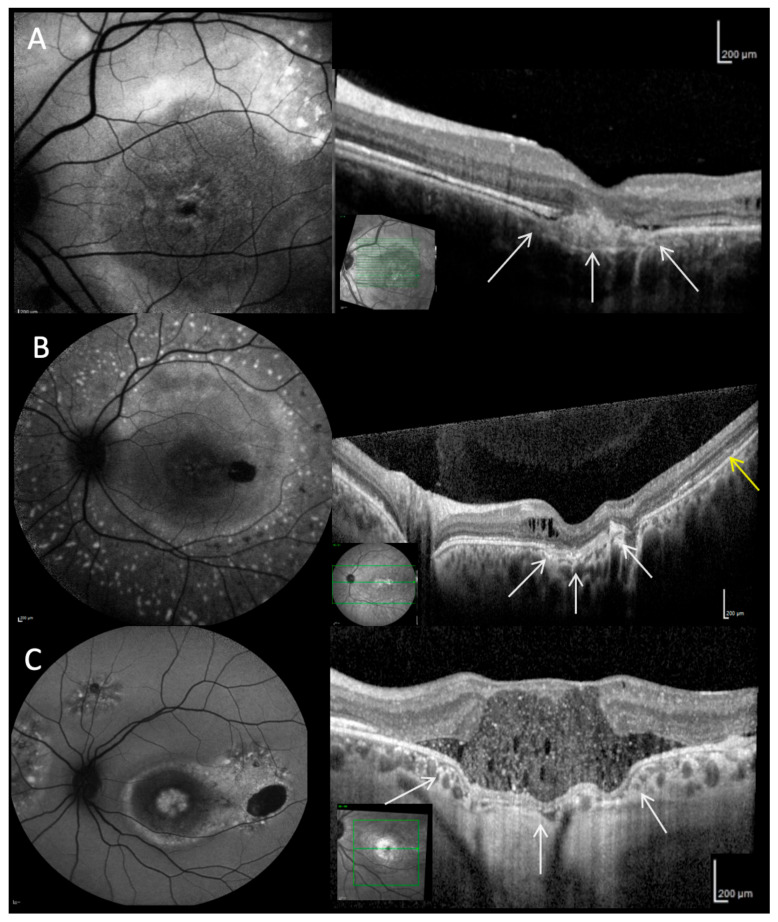
Focal and large choroidal excavation. (**A**) FAF 30° image shows confluent subretinal deposits with increased intensity arranged concentrically around the fovea (#2). OCT shows focal choroidal excavation (white arrows), a large lesion below the RPE/Bruch’s membrane complex subfoveally. Temporally, few intraretinal cystoid lesions are visible. (**B**) FAF 55° image shows multiple apparent spots with increased intensity surrounding the vessel arcades in a starry sky-like appearance (#8). The wide-field OCT shows foveal atrophy of the inner and outer retinal layers, intraretinal cystoid lesions in the papillomacular area, and focal choroidal excavation (white arrows). Temporally, a thickening of the outer retinal layers (yellow arrow) is visible. (**C**) FAF 55° image shows a lesion with reduced intensity in the macula and multiple regions with increased intensity in the mid-peripheral retina (#18). The OCT image shows a very large choroidal excavation (white arrows), associated marked thinning of the inner retinal layers and in between a large cystoid space with highly reflective material.

Various alterations in different retinal layers were identified by OCT imaging in all phenotypes (Figure 2, Figure 3 and Figure 4). The inner retinal layers (outer plexiform to nerve fiber layer) were normal bilaterally only in eight patients (#1, #2, #5–7, #12, #16, #17). The thickness of the inner retinal layers was reduced in 12/36 eyes and hyperreflective foci were present in 13/36 eyes. Mild to severe intraretinal cystoid spaces were observed in 21/36 eyes, in 10 eyes involving the fovea. In the majority of eyes (27/36) the ellipsoid zone was disintegrated in at least one area. Serous retinal detachment involving the fovea was the most common finding (32/36) varying in lateral extension as well as the height of detachment between patients and during the course of the disease (Figure 3). In most eyes with serous retinal detachment hyperreflective material (most likely elongated photoreceptor outer segments) was present adjacent to the detached retina (29/32), similar material was seen in one additional eye with probably resolved serous retinal detachment. This subretinal material was mostly continuous but could also appear as separate small stalactites in some eyes. Additional subretinal hyperreflective material adjacent to the RPE presented as bumps of variable number, size and height in 25/36 eyes. Focal choroidal excavation was seen in 10/36 eyes (Figure 4), in a single case the development could be observed over time (#2). In one additional eye choroidal excavation was more extensive with choroidal loss, marked thinning of the inner retina, and in between a large cystoid space with highly reflective material (#18). Secondary epiretinal membrane formation was seen in some patients (#9, #15) as well as a lamellar macular hole, which developed and spontaneously regressed in one patient (#18).

### 2.6. Fluorescein Angiography

Fluorescein angiography was performed only in patient #7. Late staining of regressed choroidal neovascularisation (CNV) was observed bilaterally, whereas mild to moderate increased hyperfluorescence with some flecks of marked hyperfluorescence were seen in the macula.

### 2.7. Electrophysiology

The clinical diagnosis of ARB or at least of an inherited retinal dystrophy subsequently initiating molecular genetic testing was mostly based on the retinal imaging findings. Visual field testing in a subset of eight patients (#2, #4, #9, #12, #13, #15–17) showed normal outer borders and paracentral to central scotomata. A subset of ten patients underwent electrophysiological examinations. The EOG showed an absent or reduced light rise (#1, #5, #6, #11, #13). Full-field ERG recordings were normal in two young patients (#1, #5), but showed moderately (#10) or markedly (#14, #15, #18) decreased a- and b-wave amplitudes at dark and light adaptation in four older patients. In the latter four patients and patient #2, mfERG amplitudes were centrally reduced as well. The decline of electrophysiologic responses with increasing age has been observed previously [18].

### 2.8. Molecular Genetics

**Figure 5:**
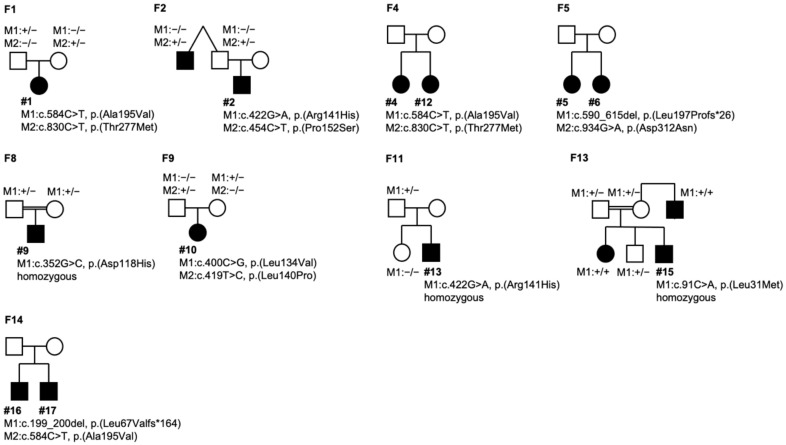
Schematic representation of selected family pedigrees. Ten pedigrees are shown for families where additional family members were available for DNA testing. *open symbols*, clinically unaffected; *solid symbols*, clinically affected. M, mutation, +/+, homozygous, +/− heterozygous, −/− wildtype).

Genetic testing of the *BEST1* gene identified biallelic mutations in each patient (Table 2). Compound heterozygosity could be confirmed by testing both parents in patients #1 and #10 (F1 and F9) and supported by the analysis of one parent for patients #2 (F2) and #13 (F11) (Table 2 and Figure 5). In addition, affected siblings in another three families (F4, F5, F14, Table 2 and Figure 5) were shown to carry two heterozygous *BEST1* mutations while both parents were asymptomatic, thus supporting a recessive mode of inheritance. Homozygosity could be confirmed by testing both parents for patients #7 (F6), #9 (F8), and #15 (F13) (Table 2 and Figure 5). Of the 16 distinct mutations, 11 represent missense mutations, four are frameshifts and one is an in frame deletion of four amino acids. Six of the sequence variations have not been reported before and were absent from control populations (gnomAD v. 2.1.1; Table S1). One of them, c.454C>T/p.(Pro152Ser), affects a codon that is known to harbor a similar ARB-linked mutation, p.(Pro152Ala) [6]. The other novel missense mutation, c.620T>A/p.(Leu207His) was found to be homozygous in patient #8. Using algorithms of three different bioinformatic prediction programs the c.620T>A/p.(Leu207His) was consistently suggested to be disease-causing. Mutation c.287_298del/p.(Gln96_Asn99del) affects the intracellular region C-terminal of transmembrane domain 2 of *BEST1*. Based on the classification of *BEST1* mutations introduced by Milenkovic et al. [19], this mutation as well as the missense-mutations belong to class 2 mutations that severely affect protein structure leading to early proteasomal degradation in the endoplasmic reticulum. In contrast, the novel frameshift mutations, c.199_200del/p.(Leu67Valfs*164), c.524del/p.(Ser175Thrfs*19) and c.590_615del/p.(Leu197Profs*26) are presumed to be null mutations.

## 3. Discussion

The ARB was first described as a new entity in 2008 [6]. Since then, clinical and/or genetic findings from about 310 ARB patients with confirmed mutations in the *BEST1* gene were described in over 65 publications. Most of the reports included only one or few patients or families, only few included more than 10 families [15,18,21,22]. In the present study, we add extensive clinical and genetic findings from a further 15 ARB families with a total of 18 ARB patients. The multitude of reports in the last decade implies that ARB may be more frequent than previously thought. The underestimation could be due to misdiagnosis of ARB [14,15,23]. In a previous study, 35.3% of ARB patients with retinal lesions were misdiagnosed as other retinal disorders and in 58.8% of ARB patients with angle-closure glaucoma retinal findings of ARB were overlooked [15]. Similarly, in the present study 7/18 patients (38.8%) were misdiagnosed as toxoplasmosis scar (#18), uveitis (#7, #9), X-linked retinoschisis (#9, after uveitis was ruled out), macular dystrophy other than ARB (#10), unknown retinal disorder (#11) or central serous chorioretinopathy (#17). Two patients were referred after they unsuccessfully underwent multiple intravitreal injections in one or both eyes based on the diagnosis of cystoid macular edema due to uveitis (#9) or exudative macular disease (#14). In addition, our study confirms the high variability of disease onset between 3 and 50 years of age, which has been reported within a range of 10–40 and 2–54 years of age in previous studies [16,24].

The most prevalent phenotype of ARB is characterized by hyperopia, yellow deposits at the posterior pole or beyond the vascular arcades, serous retinal detachment, and intraretinal cystoid spaces. The clinical course can be complicated by CNV or angle-closure glaucoma [24]. These findings are similar to phenotype I in this study, although only one of our patients had a spontaneously regressed CNV and only one patient was treated for angle-closure glaucoma. The typical ARB phenotype has been reported with a multitude of different mutations [6,14,15,16,17,21,24,25,26,27,28,29,30,31,32,33] similar to phenotype I in our cohort.

Phenotype II with involvement of the retina towards the far periphery has not been described in detail previously, which may be due to the fact that wide-angle imaging was performed only in few studies and peripheral retinal alterations might be neglected in some reports due to the more prominent central changes. One patient homozygous for c.908A>G/p.(Asp303Gly) was described having a severe retinal dystrophy [14] and members of a family homozygous for c.418C>G/p.(Leu140Pro) were initially reported having progressed retinal dystrophy with pigmentations and attenuated vessels [28]. This family has recently been re-classified as ARB [16]. Patients in other publications, e.g., patients carrying a c.422G>A/p.(Arg141His) or a c.37+1G>T mutation [28] most likely fit into phenotype II.

Phenotype III has not been described previously. The predominance of the lesion in the mid-periphery associated with the highest rod density can explain the night blindness in this patient. The patient of Tunisian origin is homozygous for a c.91C>A/p.(Leu31Met) missense mutation. This mutation has been reported in two patients of a consanguineous Tunisian family (presumably related to our patient), who presented with phenotype I [29]. Two daughters and a brother (heterozygous for c.91C>A) of the patient could be examined. The daughters had no ARB-like alterations, but the brother showed small yellow flecks in one eye. In addition, genetic testing showed that the sister of patient #15, a maternal uncle, and the daughter of that uncle were homozygous for c.91C>A. Reported macular OCT images appeared to be consistent with phenotype I, however, as the patients could not be examined peripheral changes could neither be verified nor excluded.

Phenotype IV resembling monofocal BVMD has been observed with other mutations compared to our patient in two family members of a Lebanese family harboring the compound heterozygous mutations c.209A>G/p.(Asp70Gly) and c.1403C>T/p.(Pro468Leu) [17]. A different member of that consanguineous family carries a homozygous c.209A>G/p.(Asp70Gly) mutation and presents with phenotype I.

Phenotype V resembling multifocal BVMD has been observed as well with other mutations compared to our patients in two Lebanese families harboring homozygous c.830C>T/p.(Thr277Met) or c.1403C>T/p.(Pro468Leu) mutations [17].

Four phenotypes (except phenotype III) have been described previously associated with different mutations in the *BEST1* gene, therefore a genotype-phenotype-correlation appears unlikely in ARB.

Additional clinical findings were reported such as a single patient with macular hole [30], one patient with unilateral disease [31], or optic disc drusen [29] but were not observed in the present study. We add a spontaneously regressed lamellar macular hole to the rare findings observed in ARB. Especially, we extend on a previous report of focal choroidal excavation in two ARB patients [14]. Focal or large choroidal excavation was observed in 11 eyes of nine patients or 30.6% of all eyes in the present study, which is much higher compared to 14.3% in a recent study [18]. In contrast to the latter study, especially small focal choroidal excavation, as seen in most of our patients, was neither associated with choroidal neovascularization, pigment epithelial detachment or subretinal hyperreflective material (Figure 4B). Focal choroidal excavation has been reported in up to 20% in BVMD occurring in the late stages [32,34,35] and appears to be a frequent feature of ARB as well. A large choroidal excavation as seen in our patient #18 has not been reported previously. The frequency of focal choroidal excavation depends on the density of OCT scans, as small focal choroidal excavations are often seen only on two neighboring scans with a scan density ≤188 µm.

Recently, peripapillary sparing has been described as a characteristic feature of ARB [18,33]. This was not a homogeneous finding in the present series, but most typical for phenotype II. When lesions approached the optic disc sufficiently close to define peripapillary sparing, in phenotype I 6/19 eyes and in phenotype II 6/8 eyes showed indeed peripapillary sparing. In phenotype III lesions surrounded the optic disc. The smaller lesions in phenotypes IV and V were too distant from the optic disc to define peripapillary sparing.

Hyperreflective foci, detected in OCT scans, have been described in several retinal disorders [36,37]. All patients with phenotype II (7/8 eyes) had hyperreflective foci in the inner retinal layers, as well as the patients with phenotype III (#15) and IV (#11) bilaterally. Only one patient with phenotype I (2/19 eyes) presented with hyperreflective foci. Phenotype V showed absence of hyperreflective foci. Hyperreflective foci were seen in 13/36 eyes but only in patients of 20 years of age or older.

Although a multitude of *BEST1* mutations (*n* = 348) have so far been reported to be associated with ARB and BVMD, we identified another 6 novel mutations. The majority of our patients had missense mutations. In 10 pedigrees we were able to obtain DNA samples from additional family members allowing in 7 families to unambiguously confirm recessive/biallelic inheritance of the identified mutations. In families F4, F5, and F14 only two affected siblings but not the parents were available. For these and the additional 5 isolated cases of our study the biallelic nature of the identified mutations could formally not be demonstrated. It should be noted that there still remains the remote possibility that the two identified mutations in these 11 ARB index patients exist in cis position with the consequence that these mutations would not explain the recessive phenotype. So far, however, complex alleles in autosomal recessive *BEST1* have not been reported [22,27].

In conclusion, diagnosis of ARB is challenging as the clinical presentation is highly variable even within families. Four different phenotypes could be defined consistent with previous reports and a novel fifth phenotype was described. Detailed retinal imaging including macular and wide-angle photography, FAF, and OCT are important to define ARB clinically and to initiate focused molecular genetic testing. Early detection of ARB becomes more important as new therapeutic options are developed [38].

## 4. Materials and Methods

Included in this study were patients seen at four different centers in Germany, in whom the tentative clinical diagnosis of ARB was confirmed by molecular genetic testing via chain-terminating dideoxynucleotide Sanger sequencing. The research adhered to the tenets of the Declaration of Helsinki (10/2013) and informed consent was obtained from all patients or, in the case of minors, from the parents.

### 4.1. Ophthalmological Examination

All patients underwent complete eye examination including best-corrected visual acuity (BCVA; Snellen chart), slit-lamp biomicroscopy, and ophthalmoscopy. Fundus photography was obtained with either digital fundus photography, Optos^®^ wide-angle two-wavelengths reflectance imaging (Optos Plc, Dunfermline, Scotland), or three-wavelengths reflectance imaging (MultiColor^®^, Heidelberg Engineering, Heidelberg, Germany). Fundus autofluorescence (FAF, 486/488 nm excitation) and near-infrared reflectance images (NIR, 815 nm) were obtained with a Heidelberg Retina Angiograph HRA1 or SPECTRALIS^®^ HRA (Heidelberg Engineering, Heidelberg, Germany) as described in detail previously [39,40]. Optical coherence tomography (OCT) was obtained with SPECTRALIS^®^ Spectral domain-OCT (Heidelberg Engineering) except for patient #8 (Swept-source OCT, Zeiss Plex Elite 9000^®^, Carl Zeiss Jena, Jena, Germany). In patient #9 only time-domain OCT (Stratus^®^ OCT3, Carl Zeiss Jena, Jena, Germany) was available.

In a subset of patients, kinetic Goldmann perimetry or electrophysiological examinations were performed. Full-field electroretinography (ERG), multifocal ERG (mfERG), and electro-oculography (EOG) were performed according to the standards of the International Society for Clinical Electrophysiology of Vision (ISCEV) [41,42,43] using the RETIport32 or RETIscan system (ROLAND CONSULT, Brandenburg a. d. Havel, Germany).

### 4.2. Molecular Genetic Analysis

Informed consent according to the German Genetic Diagnostics Act, was obtained by the patients or their parents prior to taking blood samples. For DNA extractions, ethylenediaminetetraacetic acid (EDTA) peripheral blood samples were obtained from all patients and some family members. Molecular genetic testing of the *BEST1* gene was done for all exonic sequences and the immediately flanking regions by the chain-terminating dideoxynucleotide Sanger methodology as described elsewhere [7]. PCR products were analyzed by direct sequencing with the BigDye terminator kit 1.1 (Applied Biosystems, Weiterstadt, Germany) following the manufacturer’s instructions. The sequencing products were analyzed on an automated capillary sequencer 3130xl Genetic Analyzer (Applied Biosystems, Weiterstadt, Germany) and evaluated with the software package Sequencing Analysis v. 5.2 (Applied Biosystems, Weiterstadt, Germany). Variants were classified as recommended by the American College of Medical Genetics and Genomics (ACMG) standards and guidelines and the Association for Molecular Pathology (AMP) Clinical Practice Guidelines [20].

## Figures and Tables

**Table 1:** Clinical findings.

Case ID	Family ID	Age (First Visit; y)	Sex	Family Members Affected	Age at Onset (y)	Follow-Up (y)	Symptoms	Right (OD) or Left (OS) Eye	Phenotype	BCVA (First Visit)	BCVA (Last Visit)	Refraction (D)
1	F1	3	F	None	3	2	Photo-phobia	OD	I	0.3	0.5	+5.00/−1.25
								OS	I	0.5	0.6	+4.25/−1.00
2	F2	7	M	Paternal uncle (BVMD)	3	5	blurred vision	OD	I	0.7	0.4	−1.00/−0.75
								OS	I	0.1	0.25	±0.00/−0.75
3	F3	7	M	None	4	0	blurred vision	OD	I	0.3	-	+3.50/−1.00
								OS	I	1.0	-	+2.50/±0.00
4	F4	7	F	Sister (#12)	6	21	blurred vision	OD	I	1.0	1.0	+0.25/−1.50
								OS	I	1.0	1.0	+0.75/−1.75
5	F5	8	F	Sister (#6)	8	2	none	OD	I	1.0	1.0	+3.75/−1.25
								OS	I	1.0	1.0	+3.75/−1.25
6	F5	9	F	Sister (#5)	9	2	none	OD	V	1.0	1.2	+1.00/−0.50
								OS	V	1.0	0.8	+0.75/−0.75
7	F6	10	F	None	10	0	blurred vision	OD	I	0.2	-	+3.75/−0.50
								OS	I	0.4	-	+4.00/−1.00
8	F7	20	F	None	12	4	blurred vision	OD	II	0.4	0.4	+2.50/−0.50
								OS	II	0.25	0.16	+3.00/−0.25
9	F8	21	M	None	4	5	blurred vision	OD	II	0.5	0.4	+0.25/−2.00
								OS	II	0.4	0.3	−0.25/−1.00
10	F9	25	F	None	17	7	blurred vision, night blindness	OD	I	0.5	0.2	+1.00/−0.75
								OS	I	0.6	0.2	+1.00/±0.00
11	F10	25	M	None	6	26	blurred vision	OD	IV	0.8	0.125	−3.25/−0.25
								OS	IV	0.8	0.2	−1.75/−1.00
12	F4	26	F	Sister (#4)	6	5	none	OD	I	1.0	1.0	±0
								OS	I	1.0	1.0	±0
13	F11	28	M	None	6	5	blurred vision	OD	II	0.16	0.1	−1.00/−1.00
								OS	II	0.1	0.05	−1.25/−0.75
14	F12	30	M	None	26	1	blurred vision	OD	II	0.3	0.25	+4.50/−3.25
								OS	II	0.08	0.05	+6.00/−3.00
15	F13	33	M	Sister, maternal uncle, brother (U)	10	2	night blindness	OD	III	0.05	0.08	+0.75/±0.00
								OS	III	0.02	0.1	+2.25/−0.50
16	F14	46	M	Brother (#17)	43	0	blurred vision	OD	V	0.6	-	+4.00/−1.25
								OS	I	0.2	-	+4.25/−0.75
17	F14	50	M	Brother (#16)	50	10	blurred vision	OD	I	0.5	0.3	+3.25/−0.50
								OS	I	0.5	0.4	+4.00/−1.75
18	F15	50	F	Brother (MD)	6	5	blurred vision	OD	V	0.1	0.125	+1.5/−0.50

BVMD: best vitelliform macular dystrophy; MD: macular dystrophy, U: unknown clinical presentation; F: female, M: male, y: years, BCVA: best corrected visual acuity, FU: follow-up.

**Table 2:** Genetic data from *BEST1* DNA testing.

Case ID	Age	Sex	Phenotype Classification	Family	Variant (NM_004183.4)	Protein	Reference (PMID)	Classification ^1^
1	3	F	I	father, heterozygous	c.584C>T	p.(Ala195Val)	10798642	5
				mother, heterozygous	c.830C>T	p.(Thr277Met)	25474345	4
2	7	M	I	mother n.a.	c.422G>A	p.(Arg141His)	18179881	5
				father/uncle heterozygous	c.454C>T	p.(Pro152Ser)	novel	4
3	7	M	I	parents n.a.	c.422G>A	p.(Arg141His)	18179881	5
					c.584C>T	p.(Ala195Val)	10798642	5
4	7	F	I	Sister #12	c.400C>G	p.(Leu134Val)	17287362	4
				parents n.a.	c.422G>A	p.(Arg141His)	18179881	5
5	8	F	I	Sister #6	c.590_615del	p.(Leu197Profs*26)	novel	5
				parents n.a.	c.934G>A	p.(Asp312Asn)	18179881	5
6	9	F	V	Sister #5	c.590_615del	p.(Leu197Profs*26)	novel	5
				parents n.a.	c.934G>A	p.(Asp312Asn)	18179881	5
7	10	F	I	*mother* heterozygous	c.620T>A	p.(Leu207His)	novel	4
				father heterozygous	c.620T>A	p.(Leu207His)	novel	4
8	20	F	II	parents n.a.	c.287_298del	p.(Gln96_Asn99del)	novel	4
				-	c.287_298del	p.(Gln96_Asn99del)	novel	4
9	21	M	II	mother, heterozygous	c.352G>C	p.(Asp118His)	32141364	4
				father, heterozygous	c.352G>C	p.(Asp118His)	32141364	4
10	25	F	I	mother, heterozygous	c.400C>G	p.(Leu134Val)	17287362	4
				father, heterozygous	c.419T>C	p.(Leu140Pro)	10798642	4
11	25	M	IV	parents n.a.	c.584C>T	p.(Ala195Val)	10798642	5
				-	c.584C>T	p.(Ala195Val)	10798642	5
12	26	F	I	Sister #4	c.400C>G	p.(Leu134Val)	17287362	4
				parents n.a.	c.422G>A	p.(Arg141His)	18179881	5
13	28	M	II	mother n.a.	c.422G>A	p.(Arg141His)	18179881	5
				father, heterozygous	c.422G>A	p.(Arg141His)	18179881	5
14	30	M	II	parents n.a.	c.346_355dup	p.(Glu119Glyfs*116)	29781975	5
					c.524del	p.(Ser175Thrfs*19)	novel	5
15	33	M	III	Mother/father/sister/brother, heterozygous	c.91C>A	p.(Leu31Met)	31254423	4
				uncle, homozygous	c.91C>A	p.(Leu31Met)	31254423	4
16	46	M	V/I	Brother #17	c.199_200del	p.(Leu67Valfs*164)	novel	5
				parents n.a.	c.584C>T	p.(Ala195Val)	10798642	5
17	50	M	I	Brother #16	c.199_200del	p.(Leu67Valfs*164)	novel	5
				parents n.a.	c.584C>T	p.(Ala195Val)	10798642	5
18	50	F	V	parents n.a.	c.140G>A	p.(Arg47His)	10854112	4
					c.422G>A	p.(Arg141His)	18179881	5

^1^ Variants were classified based on the recommendations of the American College of Medical Genetics and Genomics (ACMG) standards and guidelines and the Association for Molecular Pathology (AMP) Clinical Practice Guidelines [20]: pathogenic, 4, likely pathogenic. PMID, PubMed Identifier. n.a., not available.

## Supplementary Materials

Supplementary Materials can be found at https://www.mdpi.com/1422-0067/21/24/9353/s1.

## Abbreviations

ADVIRC	Autosomal Dominant Vitreo-Retinochoroidopathy
ARB	Autosomal Recessive Bestrophinopathy
BCVA	Best-corrected visual acuity
BVMD	Best vitelliform macular dystrophy
CNV	Choroidal neovascularization
EOG	Electro-oculography
ERG	Full-field electroretinography
FAF	Fundus autofluorescence
mfERG	Multifocal electroretinography
MRCS	MRCS syndrome (microcornea, retinal dystrophy, cataract, posterior staphyloma)
OCT	Optical Coherence Tomography
PCR	Polymerase chain reaction
RPE	Retinal pigment epithelium
