# Supplementary material for: Clinical Heterogeneity in Autosomal Recessive Bestrophinopathy with Biallelic Mutations in the BEST1 Gene
Source: Int J Mol Sci. 2020 Dec 8;21(24):9353. doi: 10.3390/ijms21249353 (PMC7763028; doi:10.3390/ijms21249353)
Supplement: Supplementary file 1 [file ijms-21-09353-s001.pdf]

**Supplemental Table S1: Classification of novel *BEST1* gene mutations**

| **Variant (NM_004183.4)** | **Protein** | **MAF (gnomAD v2.1.1)** | **Bioinformatics** | **Classification** |
|---|---|---|---|---|
| c.199_200del | p.(Leu67Valfs*164) | - | - | 5 |
| c.287_298del | p.(Gln96_Asn99del) | - | - | 4 |
| c.454C>T | p.(Pro152Ser) | - | Align GVGD (v2007): C0 (GV: 353.86 - GD: 0.00)<br>SIFT (v6.2.0): Deleterious (score: 0, median: 2.92)<br>MutationTaster (v2013): disease causing (prob: 1)<br>PP2 HumVar: probably damaging with a score of 1.000 | 4 |
| c.524del | p.(Ser175Thrfs*19) | - | - | 5 |
| c.590_615del | p.(Leu197Profs*26) | - | - | 5 |
| c.620T>A | p.(Leu207His) | - | Align GVGD (v2007): C0 (GV: 248.74 - GD: 67.97)<br>SIFT (v6.2.0): Deleterious (score: 0.02, median: 2.92)<br>MutationTaster (v2013): disease causing (prob: 1)<br>PP2 HumVar: probably damaging with a score of 0.997 | 4 |
